# Perception and Attitude of Dental Students Towards Geriatric Care: A Questionnaire-Based Survey

**DOI:** 10.7759/cureus.67297

**Published:** 2024-08-20

**Authors:** Swarnim Singh, Rekha Gupta, Shubhra Gill, Zeba Naaz, Meshi Longdo, Aarzoo Pathak, Jeldi Kusuma

**Affiliations:** 1 Department of Prosthodontics, Maulana Azad Institute of Dental Sciences, New Delhi, IND

**Keywords:** geriatric patient management, geriatric rehabilitation, delhi-ncr, postgraduate dental students, undergraduate dental students, elderly care, dental education, geriatric dentistry

## Abstract

Introduction

Geriatric dentistry concerns the influence of aging and systemic disorders on oral health. Every dental practitioner must comprehensively understand the oral health conditions affecting geriatric patients and the physiological, psychological, and social aspects of aging to deliver holistic care.

Methods

A cross-sectional survey was undertaken among postgraduate students, interns, and undergraduates in dental colleges across the Delhi National Capital Region (NCR), India. This survey employed a self-administered questionnaire to gauge the attitude of undergraduate and postgraduate students towards elderly patients.

Results

The study included 312 participants with a mean age of 24.95 years (±2.95 years). The distribution of participants by educational level: Intern: 25.3% (n=79), postgraduate (PG)-1 Year: 16.7% (n=52), PG-2 Year: 9.0% (n=28), PG-3 Year: 9.9% (n=31), undergraduate (UG)-3 Year: 19.2% (n=60), UG-4 Year: 19.9% (n=62). Most respondents strongly agreed on the necessity of being more empathetic and attentive when treating geriatric patients. Similarly, the majority strongly concurred on the differences in treatment and communication required for geriatric patients, underscoring the need for specialized training. Significant differences were observed between undergraduate (UG) and postgraduate (PG) students regarding their confidence in treating geriatric patients, their understanding of geriatric patients' expectations, and their willingness to treat geriatric patients in their future practice, with PG students agreeing on all these aspects.

Conclusion

Overall, most participants showed a favorable attitude towards elderly patients. However, postgraduates demonstrated a deeper comprehension of the needs of geriatric patients and exhibited greater confidence in their ability to treat them both presently and in future practices. Despite this, there was a recognized need for specialized training. Consequently, there is a call for increased workshops and training sessions focused on geriatric patients.

## Introduction

Global life expectancy rose 6.52 years from 66.8 years in 2000 to 73.3 years in 2019 [1]. In India, it increased by 8.68 years to 70.8 years, earning it the status of an "ageing nation" with 7.7% of citizens over 60. Improved healthcare services led to declining fertility and mortality rates, driving the demographic transition with greater reductions in mortality than fertility [2].

Geriatric dentistry addresses the impact of aging and systemic disorders on oral health [3]. A comprehensive understanding of oral health conditions in geriatric patients along with physiological, psychological, and social aspects of aging is necessary for every dental practitioner to provide holistic care to geriatric patients. Effective communication, confidence-building, and respecting elderly patients' preferences enhance patient satisfaction [4]. Continuous education on evolving geriatric care practices prepares students for such compassionate assistance.

To instill responsibility in caring for the elderly, dental students require an upbeat attitude. Evaluating the adequacy of geriatric dentistry in the curriculum is crucial for graduates' confidence in handling elderly patients [5]. Hence the questionnaire assesses undergraduate and postgraduate students' attitudes toward geriatric patients, perceptions of curriculum suitability, and confidence in treating geriatric patients.

## Materials and methods

Study design and setting

The cross-sectional study was conducted among undergraduate and postgraduate dental students in four colleges in the Delhi-National Capital Region (NCR), India. The survey was undertaken between January 2023 and June 2023.

Study population and sampling

The study population included undergraduates (3rd year, 4th year, and interns) and postgraduate students (1st, 2nd, and 3rd year) who were willing to participate from dental colleges of Delhi-NCR region, India.

The estimated sample size for the study was found to be 327 and 212 for a statistical power of 90 and 85, respectively, using the sample size calculator provided by G-Power software (version 3.0; Heinrich-Heine-Universität, Düsseldorf, Germany). The significance level (α) was set at 5%.

The questionnaire was shared among the undergraduate and postgraduate students at the institute where the study was conducted, and the snowball sampling technique was employed. The students were asked to share among their peers in dental colleges in Delhi-NCR. The questionnaire was prepared in electronic format and was subsequently disseminated using WhatsApp and email. The participation was completely voluntary, and responses were anonymous. The students were informed by proper communication channels beforehand and were requested to participate. All efforts were made to ensure that maximum participation was achieved. Two subsequent reminders to participate were given at intervals of four weeks each following the initial invitation. The hyperlink to the questionnaire remained accessible for six months.

Data collection tool and technique

Upon conducting a thorough review of the existing literature, the questionnaires included in this study were meticulously devised. The questionnaire form's consistency and reliability were validated during a pilot testing phase that included the participation of 32 dental students and Cronbach’s alpha value of 0.712 was obtained. The questionnaire's pertinence to the survey's subject matter was verified by faculty members from the Department of Prosthodontics of the institute where the study was held. 

A web-based self-administered questionnaire, consisting of two parts, was disseminated in English. The questionnaire encompassed a concise elucidation of the study's objective along with digital consent encrypted in the form. The first part of the questionnaire consisted of questions about the socio-demographic attributes of the participants such as age, gender, and year in their respective course. The next part of the questionnaire comprised a series of 12 close-ended questions regarding their opinion on geriatric population treatment, the current geriatric curriculum, and the need for specific training in geriatric dentistry. The scoring of the second part of the questionnaire was based on a 5-point Likert scale and consisted of the following options: Strongly Disagree, Disagree, Undecided, Agree, Strongly Agree (Scores from 1 to 5)

The questionnaire was sent to 350 undergraduate and postgraduate students, out of which 312 responded. The response rate of the study was 89.14%.

Ethical considerations

Institutional Human Ethics Committee clearance was obtained. Participation in the study was voluntary and there was no consequence for either participation or withdrawal. Informed consent was obtained from those willing to participate and all participants were assured confidentiality of the data.

Statistical analysis

Data was entered into a Microsoft Excel spreadsheet (Microsoft Corporation, Redmond, USA) and was checked for any discrepancies. Summarized data was presented using tables and graphs. The data was analyzed by SPSS (21.0 version; IBM Corp., Armonk, USA). The quantitative data were expressed in frequency and percentages. The chi-square test was used to compare differences in frequency across groups. A p-value of < 0.05 was considered significant.

## Results

**Figure 1:**
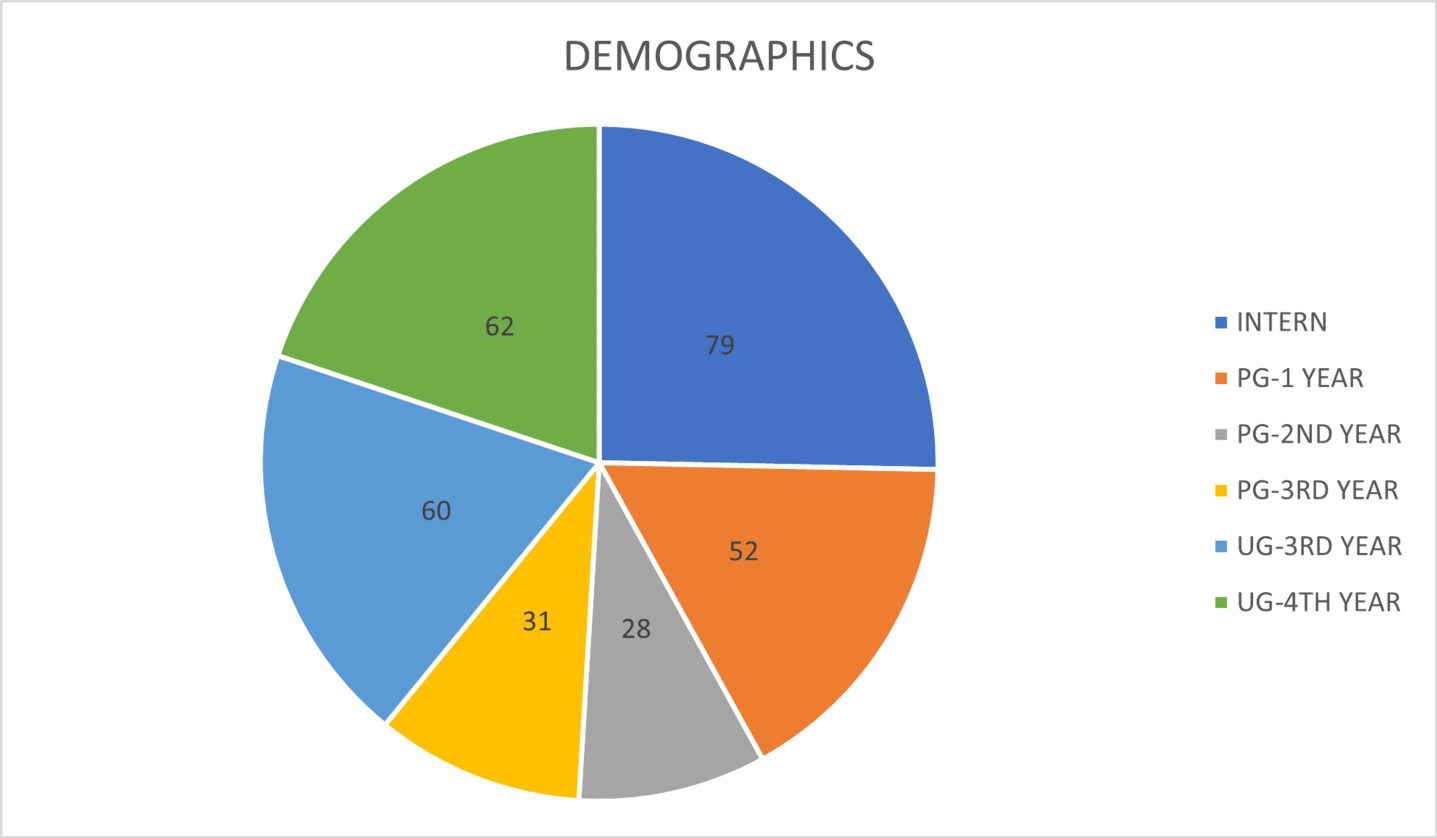
Demographics according to educational level UG = Undergraduate students; PG = Postgraduate students Intern: 25.3% (n=79), PG-1 Year: 16.7% (n=52), PG-2 Year: 9.0% (n=28), PG-3 Year: 9.9% (n=31), UG-3 Year: 19.2% (n=60), UG-4 Year: 19.9% (n=62).

The study included 312 participants with a mean age of 24.95 years (±2.95 years). The distribution of participants by educational level is depicted in Table 1 and Figure 1.

**Table 1:** Demographics according to educational level UG = Undergraduate student; PG = Postgraduate student

Year	Total number (n-value)	Percentage
Intern	79	25.3
PG-1 Year	52	16.7
PG-2 Year	28	9.0
PG-3 Year	31	9.9
UG-3 Year	60	19.2
UG-4 Year	62	19.9
Total	312	100.0

Table 2 compares different attitude items regarding the treatment of geriatric patients according to UG and PG’s attitudes.

**Table 2:** Comparison of responses to different items in the questionnaire between undergraduate and postgraduate students UG = Undergraduate student; PG = Postgraduate student P-value = Probability value (p-value of <0.05 = significant)

Questions	UG/PG	Disagree	Agree	P value
I am confident in treating and managing geriatric patients on my own	PG	37	83	0.001*
	UG	106	86	
Aging-related changes impact treatment procedure	PG	5	105	0.237
	UG	31	161	
Communicating with geriatric patient is different from other age group.	PG	5	105	0.051
	UG	39	153	
I am able to communicate with geriatric patients appropriately	PG	41	79	0.098
	UG	81	111	
Geriatric patients require longer appointments for a procedure.	PG	42	78	0.038*
	UG	88	104	
Meeting expectation of a geriatric patient different than other age group	PG	27	93	0.006*
	UG	70	122	
Geriatric patient tends to take more time in adapting to new treatment.	PG	26	94	0.441
	UG	39	153	
If yes, one should counsel them differently regarding the new treatment	PG	16	104	0.228
	UG	33	159	
One should be more sympathetic and attentive while treating geriatric patients.	PG	10	110	0.054
	UG	29	163	
I would like to treat geriatric patients in my upcoming professional years.	PG	19	101	0.018*
	UG	51	141	
The current dental curriculum is helping in treating geriatric patients better.	PG	70	50	0.404
	UG	108	84	
There should be a specific training on management of geriatric patients.	PG	16	104	0.025*
	UG	44	148	

**Figure 2:**
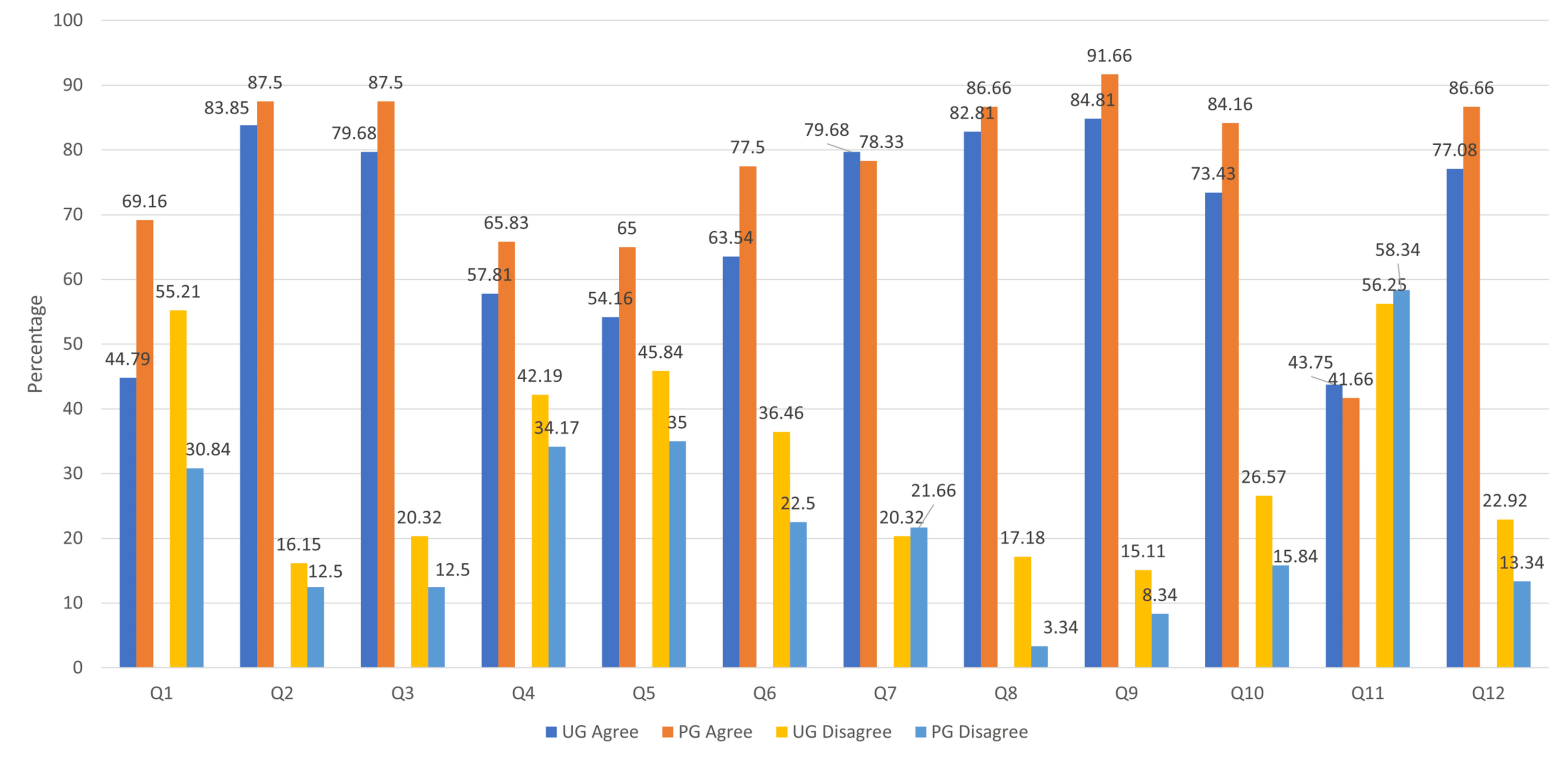
Comparison of attitude of UG and PG students to different items in the questionnaire UG agree = Percentage of undergraduate students in agreement; UG disagree = Percentage of undergraduate students in disagreement; PG agree = Percentage of postgraduate students in agreement; PG disagree = Percentage of postgraduate students in disagreement

PG students were significantly more confident in treating and managing geriatric patients on their own compared to UG students (P = 0.001). PG students were more likely to agree that geriatric patients require longer appointments for a procedure (P = 0.038), have different expectations (P = 0.006), and express a desire to treat geriatric patients in their professional years (P = 0.018). There was also significant support among PG students for specific training on the management of geriatric patients compared to UG students (P = 0.025). PG students showed a trend towards agreeing more that communicating with geriatric patients is different (P = 0.051) and that one should be more sympathetic and attentive while treating them (P = 0.054). No significant differences (P ≥ 0.10) were found between UG and PG students regarding the impact of aging-related changes on treatment procedures, the time geriatric patients take to adapt to new treatments, and whether current dental curriculum adequately prepares them for treating geriatric patients. The difference in attitude of undergraduate and postgraduate students to different items in the questionnaire is depicted in Figure 2.

## Discussion

India has acquired the label of “an ageing nation”. It is imperative to draw attention to the medical and socioeconomic issues of India's older population and investigate measures to improve their quality of life. Therefore, it is of the utmost importance to foster in the students an attitude of compassion and positivity toward the elderly to develop a sense of responsibility for providing care for them.

Elderly patients frequently have cognitive and physical impairments that might make it difficult to get comfortable dental care. Positively oriented dentists are more likely to accept these difficulties, modify their methods, and offer customized care suited to senior citizens' particular requirements and preferences [5].

Therefore, the present study was to assess the attitude of dental students toward geriatric patients and to assess the current dental curriculum in geriatric dentistry. The study showed an overall positive attitude of dental students toward geriatric patients in agreement with previous studies [6-9]. The study outcomes underscore a significant divergence in the attitudes of postgraduate (PG) students, demonstrating heightened confidence (69.1%) in independently treating and managing geriatric patients, as evidenced by a statistically significant p-value (p-value=0.001). Moreover, a notable difference emerged between undergraduate (UG) and PG students concerning the necessity for longer appointments when performing procedures on geriatric patients (p-value=0.038) and understanding the difference in expectations of geriatric patients (p-value=0.006). These results echo a prior investigation by Reuben et al., which observed a more favorable outlook correlating with increased training duration [10]. Similarly, a study conducted in Chile by Leon et al. [11] revealed a neutral perception of aging among students, while faculty members exhibited a more positive attitude, resonating with the findings of our current research.

Both UG and PG students expressed a strong consensus on the importance of being sympathetic and attentive when treating the elderly, consistent with findings from a prior study by Rejeh et al. [12]. There is a unanimous sentiment among students toward the significance of incorporating empathy and attention in their approach to the older age group, reflecting a shared commitment to addressing the unique needs of geriatric patients in their future professional endeavors.

In a study conducted in Germany by Nitschke et al. involving 463 dental students during their undergraduate education, a notable aspect involved direct interaction with institutionalized elderly individuals. The findings suggested a noteworthy shift in attitudes towards a more positive outlook, albeit with marginal changes [13].

Particularly intriguing in the present study was the discernible difference between postgraduate and undergraduate students, with postgraduates (86.6%) expressing a greater consensus on the necessity of specialized training programs (p-value=0.025). This aligns seamlessly with prior research, such as the comprehensive study conducted by Westmoreland et al. Their work accentuated the positive impact derived from exposure to an aging panel and active engagement with senior citizens. The study emphasized the compelling need for tailored training programs in elderly patient care [14]. Similarly, in the study by MacEntee et al., the brief rotation involving exposure to geriatric patients in long-term care facilities received very positive feedback from undergraduate students at the University of British Columbia [15].

A critical insight derived from the research is the significant variance in the willingness of postgraduate students (84.16%) in comparison to undergraduate students to treat geriatric patients in the future (p-value=0.025). This can be attributed to their enhanced understanding of the expectations unique to geriatric care, underlining the crucial role of specialized training in fostering competence and confidence in managing the oral health needs of the aging population.

A recent cross-sectional study conducted in Iran by Nouri et al highlights the positive attitude of dentists toward elderly patients and the need for specialized training in geriatric dentistry, advocating for the development of appropriate plans and policies to integrate such training into the dental curriculum [16]. Weber S. et al also emphasized education programs could serve as a valuable component of dental undergraduate education, addressing students' apprehensions about aging, thereby promoting positive experiences and imparting essential skills for the dental treatment of elderly patients [17]. The outcomes of this study further underscore the broader call for an evolution in dental education, reflecting the evolving demographics of patient populations and reinforcing the importance of specialized training in geriatric dentistry.

It is important to acknowledge the limitations of our study, including the potential for selection bias and the need for the replication of the study in a larger, more diverse sample. Additionally, relying on self-reported data through an online survey introduces the potential for response bias, where participants may provide socially desirable or inaccurate responses. Further investigation is warranted to gain a deeper understanding of the association between the training period and change in attitude towards the geriatric patient and how specific training would affect their attitude.

## Conclusions

Geriatric dentistry plays a crucial role in the training of future dentists, highlighting the importance of evaluating their attitudes and the existing curriculum. In the current study, the attitude of undergraduate and postgraduate students toward geriatric patients was positive but still could be improved. There was a significant difference between postgraduate and undergraduate students in terms of their confidence to treat geriatric patients, understanding of the expectations of elderly patients, and willingness to treat them in their upcoming professional years asserting years of training progressively improves the attitude. Though most students found the current curriculum on geriatric dentistry adequate, they agreed with the need for a special training program, especially postgraduate students. Therefore, there is a heightened call for more workshops and training sessions dedicated to geriatric patient care.
